# NEWS

**DOI:** 10.7189/jogh.06.020201

**Published:** 2016-12

## Africa

➢ A Department of Basic Education (DBE) report estimates that nearly 600 000 of South Africa’s disabled children do not attend school, with an estimated 5552 pupils on waiting lists for a place at a special school. A Human Rights Watch report (*Complicit in Exclusion*) published in August 2015 highlighted these problems, and calls for waiting lists to be dealt with. One of the report’s researchers, Elin Martinez, suspects that the number of children on waiting lists is underestimated, and the government does not have accurate statistics on children with disabilities who do not attend school. The system of special schools for children with severe or multiple disabilities is further strained as students with mild or moderate disabilities are referred to them rather than being accommodated in mainstream schools. This is exacerbated by the lack of support in mainstream schools, and teachers can find it very difficult to support students with disabilities due to large class sizes. Children on a waiting list for a special school will remain in their mainstream school until they are admitted, but children on a waiting list who are not already in school can find themselves excluded from both mainstream and special education, and can face lengthy waits. Disabled children can be further excluded from special schools by the inability of their parents to pay transport costs for distant schools, even if they are exempt from school fees due to low income. (*Daily Maverick*, 15 June 2016)

➢ Today’s people in Vietnam have approximately the same wealth level as Americans in the 1880s, but the same life expectancy as Americans in the 1980s. Vietnam has gained 100 years in health development, and highlights how low–income countries can convert economic growth into health improvements. It serves as an example to Africa, where many countries have lagged behind in translating economic growth into improved health. Previously Africa has been hindered by poor institutions, shoddy infrastructure and the devastating impact of HIV/AIDS. However, African countries are beginning to close the gap, and the two most basic health indicators – life expectancy and infant mortality – are improving. Prof Hans Rosling, a public health expert and co–founder of Gapminder says “it’s very clear that Africa is catching up with Europe in health … in spite of the fact that the poorest in Africa are not moving.” His research suggests that health improvements lead to lower birth rates which enable more women to work outside the home, and more resources devoted to each child –further promoting economic growth. This feedback loop is more effective if countries are well–governed, and governments invest in schools, sanitation and basic health rather than conflict. His message to African governments is that, with the right policies, health gains can be consolidated, fueling economic growth and leading Africa toward prosperity. (*Financial Times*, 8 June 2016)

➢ Following Sanofi–Pasteur’s decision to halt production of Fav–Afrique, stocks of Africa’s most effective anti–venom treatment for snake bites will expire at the end of June 2016. Each year, 30 000 African people die from snake–bites, and although other treatments are available, none are as effective as Fav–Afrique which treats bites from 10 types of snake. Sanofi–Pasteur decided to discontinue production in 2010, offering to share the technology with anyone who wanted to resume its production – to date, no–one has taken up this offer. Fav–Afrique is complicated and time–consuming to produce, expensive (at an average cost of US$ 500 per patient) and requires refrigeration – difficult for rural African clinics. The demise of Fav–Afrique reflects a wider market failure in the pharmaceutical industry; companies like Sanofi–Pasteur claim they are not making a fair rate of return on investments and call for donors, governments and other organisations to fund essential drugs like vaccines and anti–venoms. (*Voice of America*, 24 June 2016)

➢ Stigma is one of the biggest obstacles in ending South Africa’s HIV/AIDS epidemic. The HIV prevalence rate among sex workers – one of the most at–risk groups – and their clients in Durban was 53%, compared to the national average adult rate of 19.2%. Sex workers report being humiliated and dismissed by staff in health clinics, and Dr Luiz Loures, the deputy executive director of UNAIDS believes that discrimination against key populations such as sex workers and gay men means that AIDS is selectively returning despite medical advances in life–saving HIV drugs. Durban recently hosted the 16th International AIDS Conference, and campaigners who had previously fought to break the silence around AIDS are now alarmed over unequal access to HIV treatment. South Africa saw 380 000 new HIV infections and 180 000 deaths from AIDS in 2015. Mark Heywood, a leading activist from the Section 27 rights group marched to “wake up the world again”, saying that “people with HIV are mostly poor, they’re mostly marginalised.” This is a backdrop of falling international AIDS funding and only 17 million of the world’s 36.7 million HIV–positive people having access to HIV treatment. (*Reuters*, 18 August 2016)

➢ According to the WHO, more than 120 000 people die each year in Africa from fake anti–malarial drugs, which can be sub–standard or contain no active ingredients. Substandard drugs, with insufficient quantities of active ingredients, can also lead to drug resistance – particularly dangerous for diseases like malaria and tuberculosis. By some estimates, one–third of all anti–malarial drugs in sub–Saharan Africa are fake. However, some small companies are using technology to tackle the problem. For example, Sproxil, led by Dr Ashifi Gogo, offers scratch–panel stickers with an authentication code for consumers to text to Sproxil for verification. Sproxil spokesman Tolulope Gbamolayun says that 29 million verifications have taken place since the scheme launched in 2009. Bright Simons set up a similar “verify–by–mobile” system (mPedigree Network), where printed barcodes and scratch–off stickers – developed in partnership with Hewlett–Packard – help customers verify drugs. In Nigeria, the technology is helping regulators track down fraudsters. The pharmaceutical industry has also introduced a number of technologies to detect fake drugs, and the US Pharmacopeial Convention has set up a center for pharmaceutical training to train African pharmacists in screening for substandard and fake drugs. However, such initiatives have to deal with the massive scale of fake medicines, government corruption, lack of accountability, inaction from multinational pharmaceutical companies, and under–resourced drug regulatory bodies. (*BBC*, 28 September 2016)

## Asia

➢ In June, Tibetan female farm–workers marched on the capital Lhasa against the confiscation of their farmland to make way for new urban developments. According to an anonymous source, the protest was confined to women, out of fears that the Chinese police would crack down on any men taking part. The Chinese authorities had promised to compensate the farmers at a rate of US$ 30 352 per *mu* [approx. 0.16 acres] of land, but farmers claim that they have only received US$ 3035 per mu, and are demanding US$ 27 316 per *mu*. The farmers claim that confiscating their land at such low rates of compensation will drive them into poverty for generations. Other sources say that traditional Tibetan homes in the Lhandrub region are being destroyed and replaced by Chinese–style dwellings, without the consent of the citizens. (*Radio Free Asia*, 10 June 2016)

➢ Northern Myanmar’s jade mines employ an estimated 300 000 migrant workers, drawing people from across the country. Scavenging through mine waste to find jade stones is potentially lucrative, but living conditions are harsh and dangerous landslides are common. Despite the dangerous conditions, drug addiction is a bigger threat to miners’ health. There are no official statistics, but according to local activists up to 50% of miners are drug users in some areas. The miners are drawn to drugs and alcohol to ease physical pain and help with relaxation after intense manual labor. Opium, heroin and methamphetamine are readily available in many mining areas, produced by ethnic rebel groups, militias and criminal gangs. Drug users who share needles are at risk of HIV infection, and there is some provision of harm reduction services such as clean needles, methadone, HIV testing, counselling and antiretroviral drugs. However, Myanmar’s Drug Advocacy Group has called on the new NLD government to scale–up services and adopt a rights– and health care approach to the drugs problem. And despite the existence of – albeit limited – harm reduction services, law enforcement against dealers is almost non–existent, compounded by buying–off local officials, police and military officers. (*Irrawaddy*, 20 June 2016)

➢ The Association of Southeast Asian Nations (ASEAN) fully ratified its Open Skies policy in May 2016 following Indonesia and Laos sanctioning it. It means that airlines from its 10 member states can fly freely in the region. The increased connectivity and reduced prices should boost tourism – 12% of the region’s GDP – but is less helpful to dengue control. Dengue is set to join the list of diseases spread by air travel, which includes measles, influenza and SARS. With 70% of the global dengue burden, the open skies policy will probably lead to the wider spread of the disease. Severe dengue outbreaks would cause heavy losses in tourism revenue – it is estimated that Thailand alone could lose US$ 363 million if tourism from non–epidemic countries fell by a mere 4%. It is imperative that governments rely less on sporadic and individual vector control, but focus on efforts to limit transmission risk, introduce dengue vaccination programmes, and to keep a high level of public awareness to minimise the spread of dengue. (*The Diplomat*, 15 June 2016)

➢ The recent arrest of the rapper Namewee, whose video depicted religious leaders dancing in a mosque, highlights how Malaysian Islam is gradually becoming more conservative. The government, led by Mr Najib Razak, appears to be less autonomous on religious policy, more reliant on Islamic advisers, and less likely to rein in Islamic firebrands. This may be an attempt by Mr Razak and his ruling party, which nearly lost the 2013 general election, to bolster support among the Islamic opposition and distract attention from a scandal whereby billions of dollars went missing from a state–owned investment company and Mr Najib was implicated in receiving some of the money. The ruling party has fast–tracked the reading of a bill from the Pan–Malaysian Islamic Party (PAS), which would increase the punishments that Islamic courts can impose on Muslims convicted of religious offenses (currently fines, six strokes from a cane or three years in jail) – and some in PAS think that Muslims who drink alcohol should receive up to 80 lashes, and those who have sex outside marriage up to 100 lashes. This may exacerbate Malaysia’s exodus of young people, including moderate Muslims, and a rise in such rhetoric could encourage radicals. Indeed, almost 70 Malaysians have had their passports cancelled after attempting to join Islamic State, three people have been arrested after allegedly plotting attacks on nightspots and a Hindu temple, and 11% of Malaysians have a favorable view of Islamic State, compared to 4% in Indonesia. This is all much more worrying than Namewee’s videos. (*The Economist*, 24 September 2016)

➢ The number of confirmed Zika cases in Vietnam has doubled to 23 over the past 3 days, with a dozen new infections confirmed in Ho Chi Minh City. Zika has been spreading in Southeast Asia after outbreaks in the Americas, and Thailand reported the region’s first confirmed case of the birth defect microcephaly. Vietnam also reported a microcephaly case that is probably linked to Zika. In October, Vietnam raised the alert level for Zika, and stepped up the monitoring of pregnant women. There is no vaccine or treatment for Zika, and an estimated 80% of those infected report no symptoms, making it difficult for pregnant women to know whether they have been infected. As well as microcephaly, Zika has also been linked to other neurological disorders, including Guillian–Barre. (*Voice of America*, 2 November 2016)

## Australia and Western Pacific

➢ Mr Masamitsu Yamamoto, a 58–year old man, is on trial at the Tokyo District Court over his alleged violation of Japan’s Cannabis Control Law. Mr Yamamoto has terminal liver cancer, and maintains that he uses cannabis as pain relief after exhausting all other options and failing to get access to legal cannabis treatment. Some European countries, US states and Canadian provinces allow the medical use of cannabis, but Japan’s Article 4 of the Cannabis Control Law specifically bans the use or prescription of medicines derived from cannabis – and violators face up to 5 years in prison. Mr Yamamoto is campaigning for the “compassionate use” of medical marijuana. Dr Kazunori Fukuda, a former cancer researcher at the National Cancer Center Research Institute, argues that cancer patients suffer from appetite loss and depression, which can be alleviated by cannabis. However, an official from Japan’s Health, Labor and Welfare Ministry states that the WHO has not given clear guidance on the use of medical marijuana, and that cannabis, a controlled substance under international treaties, is potentially a “gateway drug” to other drugs. “We need to weigh the risks of abuse against the wishes of a few people who want to use marijuana,” the official said. (*Japan Times*, 26 June 2016)

➢ Mr Ashley Peacock, a New Zealand citizen, suffers from severe autism and psychotic episodes, and spends most of his time in an isolated mental health unit despite posing no risk to the wider public. He lives in a 3m x 4m room with a mattress on the floor and no toilet facilities, and is allowed outside for 90 minutes each day. Autism Action NZ have campaigned for his release, and argue that his case highlights shortfalls in the care of people with severe autism in New Zealand. They call for crisis teams that specialise in autism, with an awareness of autism’s co–morbidities with mental health issues, and for residential care facilities that can meet the needs of people like Mr Peacock. Without this support, people with autism either “fall through the cracks” or end up in the mental health system which cannot meet their needs, according to Kim Hall of Autism Action NZ. (*radionz.co.nz*, 8 June 2016)

➢ Australia has joined a handful of countries where AIDS is no longer a public health issue. At the peak of the Australian AIDS epidemic in the 1990s, 1000 people died each year from the illness. However, since the introduction of antiretroviral drugs in the mid–1990s, the number of Australians dying each year from AIDS is so low that numbers are not recorded. However, researchers point out that ending the AIDS epidemic does not mean that the end of HIV infections – each year, 1000 Australians are infected with HIV. There are concerns that some young people who did not witness the AIDS epidemic in the 1980s and 1990s may be complacent about the risks of HIV infection. Don Baxter, from the Australian Federation of AIDS Organisations, calls for Australia to continue to support other countries who are still dealing with the AIDS epidemic, and for the country to increase its financial support to the Global Fund to target HIV and AIDS internationally. (*ABC*, 10 July 2016)

➢ Australia and Papua New Guinea have agreed to close the controversial detention camp for asylum seekers on Manus Island, off Papua New Guinea. Asylum seekers attempting to reach Australia are sent to either Manus or Nauru, and are ineligible to be settled in Australia. These camps have faced criticism from the UN and various human rights groups, and there are reports of abuse, assault, inadequate medical care and dirty, cramped conditions. Australia has defended its use of the camps and asylum policy, saying it is necessary to prevent people dying on the dangerous crossing from Indonesia to Australia – hundreds of people have already died attempting it. Mr Peter Dutton, Australia’s immigration minister, confirmed that Australia would not accept any of the refugees held in Manus, who face either being resettled in Papua New Guinea or returned to their home country. Some asylum–seekers have spent years in the camp, and furthermore there are reports of refugees being attacked in Papua New Guinea. (*Al Jazeera*, 17 August 2016)

➢ Although Japan’s care for pregnant women is aligning with practices in other developed countries, it differs in the provision of pain relief during labour. Women are generally treated as fragile during their pregnancy, but little pain–relief is offered during labour, and Japan’s Buddhist tradition teaches that women should embrace the pain of labour to prepare for motherhood and promote bonding with their baby. Many women are keen to have an epidural anesthetic during childbirth, but few obstetric centres or hospitals provide them – and almost never outside normal working hours. The national health–insurance scheme’s contribution toward childbirth (US$ 4053) does not generally cover the cost of an epidural. Japan’s government is keen increase the country’s fertility rate from the current 1.5 children per woman to 1.8 to slow population shrinkage, and making childbirth a less painful experience may help. Another worrying trend is Japan’s high and rising proportion of underweight babies (2.5 kg or less at birth) – in 2015, 9% of babies were underweight. One reason is that women do not gain enough weight during pregnancy – doctors advise women to gain no more than 6–10 kg, compared to 11–16 kg in the UK. (*Economist*, 22 October 2016)

## China

➢ The town of Wukan (widely regarded as an incubator for grass–roots democracy in China following its residents’ eviction of local Communist officials and police in 2011 over alleged corruption) has been locked down by riot police and Secretary Lin Zulian – its chief – arrested after he called for renewed protests against corruption and land–grabs. According to the police, Secretary Lin was detained because prosecutors suspect him of having received bribes, but residents took to the streets to protest his arrest, and to call attention to the land disputes. According to one resident, the town’s residents fully believe that Secretary Lin’s arrest is the work of higher authorities who wish to suppress the [land] issue. In a draft speech circulated online to residents before his arrest, Mr Lin called for renewed protests against land corruption and for residents to sign a petition. He also called for loyalty to the Communist Party, collective democracy under the rule of law, and stated that “first, cursing and hitting people is not allowed. Second, destroying public order is not allowed. Third, smashing things is not allowed and violators will be severely punished.” The town was patrolled by several hundred riot police with shields and batons following his arrest. (*Financial Times*, 19 June 2016)

➢ According to a report from the World Bank Group, the World Health Organization and Chinese government agencies, structural reform to China’s health–care system could save up to 3% of its GDP. China needs to slow down its main drivers of health–care costs (an aging population, and soaring incidence of cancer, diabetes and heart disease), and avoid the creation of “high cost, low–value” health care. The report recommends measures such as bolstering China’s primary care system and allowing private–sector providers greater access to the public sector. The World Bank estimates that health expenditure in China will increase from US$ 529 billion in 2015 to US$ 2.35 trillion in 2035 – which is 5.6% and 9.1% of GDP respectively – without reform. China has made progress in improving health–care access, with almost its entire population covered by some form of health insurance, but the country’s public hospitals are overwhelmed, and rely on medicine sales for revenue which creates skewed incentives for doctors to over–prescribe. “We’re confident that these reforms will help China build a strong foundation to create a healthier population, which will be an engine for job creation and sustainable economic growth,” says Jim Yong Kim, the president of the World Bank Group. (*Bloomberg*, 22 July 2016)

➢ Mr Tan Jingsong, a partially–sighted law graduate, was rejected for a government job he applied for in Yueyang, Hunan province – despite receiving top marks in the written exam undertaken by all the candidates. The job did not specify any physical requirements, but Yueyang’s Human Resources Bureau rejected Tan’s application because the General Standard for the Physical Examination of Civil Servants specifies a minimum vision threshold of 0.8 in both eyes – Tan is completely blind in one eye, and has a corrected vision of 0.3 in the other. The Disabled Persons’ Federation point out that the State Council and Hunan Province have stated that disabled people should make up at least 1.5% of employees in public institutions. Chao Xiangyang, the Federation’s deputy director, says that the General Standard fails to take disabled people into account, and almost invalidates any other legal employment protections. Associate Prof. Liu Xiaonan of China University of Political Science and Law believes that the General Standard is not sufficiently job–specific, and may lead to institutional discrimination. “Disabled people in public service can better reflect the civilisation and equality of a country,” Liu says. (*People’s Daily Online*, 18 August 2016)

➢ Following a recent survey, China’s State Food and Drug Administration (SFDA) regulator found fraudulent practice on a massive scale, with more than 80% of clinical data being “fabricated”. It looked at data from 1622 clinical trial programmes of new drugs awaiting regulatory approval for mass production, and discovered that much of the data gathered during these trials was incomplete, failed to meet analysis requirements or was untraceable. This exposé was published in the *Economic Information Daily* newspaper, which also cited a source claiming that some companies were suspected of deliberately hiding or deleting records of adverse effects, and tampering with data that did not meet expectations. As a result, more than 80% of mass–production applications for new drugs have been cancelled, and further evidence of malpractice may emerge. According to a health care insider, China’s generic drugs industry is plagued with quality problems, and many “new” drugs are merely combination of existing drugs while clinical trial outcomes are written beforehand with the data massaged to fit in. Another source claimed that national standards on clinical trials are not widely implemented. The regulatory problems around China’s pharmaceuticals industry is shown elsewhere, reflected in the Chinese public’s bulk–buying of items such as infant milk formula produced overseas. (*Radio Free Asia*, 22 September 2016)

➢ China’s State Council outlined plans to increase its citizens’ average life expectancy to 79 years by 2030, from its current level of 76.34 years. Its “Healthy China 2030” blueprint outlined plans to invest in areas such as elderly care, medical equipment and data, and food safety, and to increase expenditure on health care to US$ 2.37 trillion a year, from its current US$ 0.66 trillion. The government plans to have three doctors per 1000 people, and to reduce infant mortality, traffic deaths, deaths from chronic diseases, improve psychological interventions, and reduce smoking and alcohol abuse. It also intends to improve cancer survival rates, increase physical activity and introduce a national monitoring system for food safety and food–borne diseases. The blueprint also commits to tackling China’s gender imbalance by setting up birth monitoring systems. (*Asia Times*, 26 October 2016)

## Europe

➢ Most European countries have strict laws on drug–taking, but over the past few years most have relaxed their enforcement, with recreational users being fined or warned rather than jailed. This is led by the Netherlands with its tolerance policy on the possession of cannabis, the Czech Republic which decriminalised the possession of small quantities of drugs, and Portugal which decriminalised the possession of all drugs for personal use. However, more recently the trend toward relaxation has halted, despite the Netherlands, the Czech Republic and Portugal highlighting the success of these policies. These countries illustrate that decriminalisation does not lead to higher consumption or act as a gateway to harder drugs, and that it is linked to lower HIV infections. Their drug–related fatalities also appear to be lower than other countries with stricter laws. However, even these three trail–blazing countries are tightening their approach, due in part to squeezed public finances (less money for harm–reduction services), complacency (the success of the policies means that politicians may think the problem is solved), and public backlash against liberal drugs policies. The timing of Europe’s tightening policy on drugs is unfortunate, as there are some worrying signs that the USA’s epidemic of opioid consumption may spread to Europe. (*Economist*, 18 June 2016)

➢ The High Court in England ruled that the NHS is able to fund pre–exposure prophylaxis drugs (“PReP”), which are highly effective in preventing HIV infection following transmission. When taken consistently, it reduces the risk of HIV infection by more than 90%. NHS England claimed it did not have the power to fund preventative treatments, and the National AIDS Trust (NAT) challenged this decision in court. Mr Justice Green ruled that NHS England “has erred in deciding that it has no power or duty to commission the preventative drugs in issue.” The NAT are now calling for the NHS to make PReP immediately available, pointing out that over 4000 people each year are infected with HIV in the UK, and that further prevention options are desperately needed to supplement condom use. The British Medical Association welcomed the court’s ruling, saying that PreP could “help save many lives”. However, NHS England intends to appeal the ruling, saying Mr Justice Green’s interpretation of legislation was inconsistent with Parliament’s intent. (*The Independent*, 2 August 2016)

➢ According to the National Records of Scotland, drugs–related deaths reached a record high in Scotland, with a total of 706 people dying in 2015 from drug abuse. This is a 15% increase from 2014, when 613 people died, and the number of drugs deaths in Scotland has been steadily increasing since 1995, when 426 deaths were recorded. Deaths were particularly high among older users, with 73% of deaths occurring in people aged over 35 years, while deaths among younger users (aged under 24) fell. Heroin and morphine were involved in 49% of deaths – the highest–ever level. Aileen Campbell, the minister for public health stated that drug use is falling overall in Scotland, and that the health problems of older drug–users is a legacy of historical drug abuse. However, the group Addaction Scotland highlighted falling numbers of fixed–site needle exchanges – often users’ entry points for treatment – and cuts in treatment services. Other health professionals and drug charities have called for supervised heroin consumption to enable users to receive drugs more safely. This could also reduce the number of new HIV infections; in Glasgow drug–related HIV infections rose to 47 in 2015 from an average of 10 per year, with public injecting posing further risks. (*BBC*, 27 August 2016)

➢ The latest European Social Survey (ESS), based on a survey of 40 000 people across 21 European Europe, found that a large number of European people are affecting by physical and mental health conditions. It uncovered large gender discrepancies in depression and severe headaches, as across Europe women are much more likely to report these conditions. Men are much more likely to smoke compared to women, with smoking rates much lower in northern Europe, the UK and Ireland; and considerably higher in central and eastern Europe. Overall, Sweden has the least number of smokers – 15% – a huge fall compared to its earlier rates of 77.8% and 76.2% for men and women. Men are more likely to report being overweight than woman in all countries, and the highest rates are in central and eastern Europe, and the lowest rates in Switzerland, Austria and Denmark. Binge drinking is particularly common in the UK and Portugal, but rarer in Nordic countries and among women in central and eastern Europe. Across all 21 countries, men consume almost twice as many units as women, and overall drinking rates are particularly high in Ireland. (*Science Daily*, 23 October 2016)

➢ Worldwide, stock markets fell as Donald Trump was elected as the next President of the USA. However, share prices in many European pharmaceutical companies rose sharply, with Hikma Pharmaceuticals rising 6%, Roche and Novartis rising by more than 4%, and GlaxoSmithKline rose 1.8%. These rises were driven by market expectations that a Trump presidency would end new rules controlling the price of pharmaceuticals, which had followed an earlier public outcry over drug prices (eg, Retrophin’s announcement that it was increasing the price of an anti–parastic drug from US$ 13.50 to US$ 750 per dose). The European pharmaceutical market has traditionally performed well in periods of uncertainty and a strong US dollar. Barclay equity strategists included four pharmaceutical and health care companies in their list of the 10 European countries most likely to benefit from a Trump victory, namely Actelion, Hikma Pharmaceuticals, UCB SA and H Lundbeck. Furthermore, the election of a Republican Senate could provide a further boost to the European health care sector. (*Business Insider*, 9 November 2016)

## India

➢ Médecins Sans Frontières (MSF) has warned that India will not remain “the pharmacy of the world” if proposals under the Regional Comprehensive Economic Partnership (RCEP) agreement between the 10 ASEAN countries are adopted. Currently, developing countries are exempt from “Data Exclusivity” and “Patent Term Extensions” (which offers legal protection for a drug beyond the normal patent rules and duration), but leaked proposals from the RCEP trade talks show that some countries are proposing to halt these exemptions. If implemented, India would be prohibited from registering more affordable versions of a drug while the exclusivity is in force over the clinical trial data. “We appeal to India’s IP [intellectual property] negotiators in particular to stand by the promises made last week by Health Minister JP Nadda at the UN High Level Meeting on HIV/AIDS that ‘India is committed to maintaining TRIPS [Trade Related Aspects of Intellectual Property Rights] flexibilities to ensure access to affordable medicines,” said Leena Menghaney, South Asia Head of MSF’s Access Campaign. (*The Hindu*, 19 June 2016)

➢ The Punjab is suffering a hidden epidemic of drug abuse, with nearly 20% of young men in the state using opioids. Within the past 5 years, Punjab’s drug users have increasingly switched from the traditional poppy husks toward injectionable heroin. The levels of drug misuse in the Punjab are particularly acute, and may be caused by falling agricultural employment not being off–set by job–creation in the cities. Gursharan Singh Kainth, an economist, calls for an “agro–industrial revolution” to provide better jobs for young men. Punjab’s plight is revealed in a new film, *Udta Punjab,* which draws a parallel with a real–life case of a convicted drug lord who named a leading politician’s brother–in–law as his accomplice. The film nearly missed its release date due to India’s film board demanding 89 cuts, including every reference to Punjab, but thanks to an intervention by the Bombay High Court, the censors were over–ruled. (*The Economist*, 25 June 2016)

➢ The *Bangalore Mirror* newspaper reports that an estimated 199 060 people are living with HIV in the state of Karnataka, but only 128 399 people are receiving antiretroviral treatment (ART). In addition, despite having the second–highest number of ART centers in India, Karnataka faces problems of staff shortages. The WHO recommends that treatment begins as soon as possible after diagnosis, regardless of the CD4 white cell count, but across India, ART begins when the cell count falls below 350. Even at this level, there are an estimated 1 345 678 people with CD4 counts of less than 350, but 940 000 receive treatment, giving a coverage rate of 70%. Dr Aneesha Ahluwalia, a health expert, calls for the Indian government to follow the WHO’s guidelines on ART, noting that people are less likely to come back for follow–up appointments and the difficulties in tracking the incidence of HIV under the current system. (*The Bangalore Mirror*, 19 September 2016)

➢ The Liver Foundation, a Kolkata–based charity, is training unqualified medics in primary medical care. India has one of the worst doctor–to–patient ratios in the world, which gives rise to self–trained practitioners – and recently, several children died in the Tamil Nadu state after reportedly being treated by unqualified medics. However, the Liver Foundation believes that unqualified medics should be used for health care to help bridge the serious staffing shortfall which is especially prevalent in rural areas, and a study published in *Science* aimed to assess the effectiveness of the foundation’s training program. Some unqualified practitioners reported being more confident in their roles, and overall they were more likely to adhere to checklists and provide correct treatments. However, although the rates of unnecessary drug prescriptions were unaffected by training, they did not differ significantly from publicly–trained doctors who are often poorly trained and lack incentives to provide good care. This suggests that training unqualified practitioners (who are then designated as village health workers rather than doctors) can improve their practice to match those of public sector doctors. Training is already expected to be scaled–up in Tamil Nadu, and offered to thousands more informal practitioners. This move is likely to be resisted, and previously the Indian Medical Association has taken legal action to block similar schemes. (*BBC*, 11 October 2016)

➢ India launched a new scheme to provide free health check–ups to pregnant women at government health centers and hospitals, and women living in under–served, semi–urban, poor or rural areas will be targeted. Prime Minister Modi appealed to doctors to contribute 12 days each year to save mothers and newborns’ lives in India, where 45 000 pregnant woman die each year – of which, only 19.7% benefited from pre–natal health checks. So far, 15 000 gynecologists and obstetricians have volunteered to participate, and the government is keen to introduce techniques from the private sector – where death rates are lower – into the screening program. Women will be tested for anemia, blood pressure, high blood glucose, hormonal disorders and other pregnancy–related problems, and will be provided with free ultrasounds to track their unborn baby’s health and development. (*Hindustan Times*, 4 November 2016)

## The Americas

➢ A survey published in the Journal of the American Medical Association has found some improvements in the US diet from 1999 to 2012. The percentage of people eating poor diets has fallen from 56% to a still–high 46%, and the majority of citizen still don’t eat an optimal diet. The biggest changes are in the number of people reporting higher grain consumption, and slightly higher consumption of nuts, seeds and yoghurt, and less sugary drinks, white potatoes and refined grains. However, the amount of fruit and vegetables consumed has not changed, and people are still consuming the same levels of meat, processed meat and sodium. The picture is starker when the diets of various groups are considered: white Americans improved their diets the most, while improvements among poorer people and minority groups were smaller. These groups are often the targets of aggressive marketing campaigns for junk food. Improving the country’s diet is essential for improving its overall health, and one of the study authors, Dr Dariush Mozaffarian calls for the US to address its food issues. “We have strong policies about safety for cars, about safety for toys, about safety for workplaces and offices we come to, about car seats, about seat belts. And yet we have very little strong government policy about overall healthy foods,” he says. (*Time*, 21 June 2016)

➢ Venezuela was certified free of malaria in 1961, nine years ahead of the USA. However, there has been a 356% increase in malaria cases since the 1990s, and Venezuela may have 200 000 cases in 2016. According to unofficial figures compiled by doctors, Venezuela has had125 158 infections to date in 2016, compared to 136 402 for the whole of 2015 – which was itself a 75–year high. Venezuela’s malaria outbreak also threatens neighboring Brazil and Colombia. According to the Health Ministry, only 300 mosquito nets were distributed in 2014, despite 800 000 people living in high–risk areas, and the country spends less than US $1 per person on malaria control – the second–lowest figure in Latin America. This means that the situation is likely to deteriorate; it is already compounded by drugs shortages arising from the country’s economic crisis, and people flocking to gold–mines for employment – located in malaria–prone areas – and spreading the disease when they return home. (*Fox News*, 17 August 2016)

➢ The Colombian president, Juan Manuel Santos, was awarded the 2016 Nobel Peace Prize for his “resolute efforts to bring the country’s more than 50–year–long civil war to an end.” The Colombian civil war has killed more than 220 000 people and displaced millions. The prize was awarded despite the country’s voters rejecting the peace deal between the government and the FARC rebels in a referendum shortly after the announcement. The committee states that this setback does not necessarily mean the end of the peace process, as it was a rejection of a specific peace agreement and not the desire for peace. Mr Santos pledged to continue to search for peace until the end of his mandate. “I accept it not on my behalf, but on behalf of all Colombians, especially the millions of victims of this conflict which we have suffered for more than 50 years,” said Mr Santos. (*NPR*, 7 October 2016)

➢ Following on from the legislative and technical difficulties behind the Affordable Care Act (“Obamacare”), health care reform in the USA has hit another problem, as premiums on covered plans have increased by 20%. While this appears to be a major setback for health care reform, the spike in premiums only affects the plans traded on the “exchanges” insurance market system, for people who are not covered by their employers or government programs such as Medicare. The main driver for this increase is insufficient healthy people signing up for Obamacare in many states, which has affected insurers’ risk calculations. However, most people who buy health insurance on the exchanges system are eligible for subsidies, which offers some protection and are therefore less likely to withdraw from Obamacare. Moreover, despite these setbacks, Obamacare has succeeded in reducing the number of US citizens without health insurance – now at its lowest level – and in reining in the growth of health care costs. (*Seattle Times*, 30 October 2016)

➢ Haiti has been devastated by Hurricane Matthew, the fiercest storm to hit the country in 50 years, and the latest catastrophe to hit the country which has still not recovered from the 2010 earthquake which killed 200 000 people and caused damages of US$ 8 billion – 120% of GDP. Hurricane Matthew wiped out livestock and crops such as rice, bananas and coconuts on which most people depend. Haiti is already the poorest country in the western hemisphere, and relies on US$ 2.2 billion of annual remittances from its diaspora, and more than 20% of the government’s annual budget comes from foreign aid or direct budget support. As post–hurricane efforts get under way, some people, including Maarten Boute, a prominent businessman in Haiti, are calling for a new approach to reconstruction following on from mistakes in the post–earthquake reconstruction. Mr Boute argues that the best way to help Haiti is to source relief locally, buy Haitian exports, and generate investment and tourism; and there must be a sharper focus on productive projects with Haitian partners. This is illustrated by the Caracol industrial park – intended to boost the clothing industry after 2010 – which has fallen short of its job–creation goals, and farmers who lost their land for the project complain that they were not properly compensated. “This is a long–term job. We’re not just rebuilding the hurricane damage, we’re laying the foundations for the future,” says Mr Boute. (*Financial Times*, 2 November 2016)

